# Draft Genome Sequence of Sorghum Grain Mold Fungus *Epicoccum sorghinum*, a Producer of Tenuazonic Acid

**DOI:** 10.1128/genomeA.01495-16

**Published:** 2017-01-26

**Authors:** Rodrigo C. Oliveira, Karen W. Davenport, Blake Hovde, Danielle Silva, Patrick S. G. Chain, Benedito Correa, Debora F. Rodrigues

**Affiliations:** a Department of Microbiology, University of São Paulo, São Paulo, Brazil; b Biosciences Division, Los Alamos National Laboratory, Los Alamos, New Mexico, USA; c Civil and Environmental Engineering, University of Houston, Houston, Texas, USA

## Abstract

The facultative plant pathogen *Epicoccum sorghinum* is associated with grain mold of sorghum and produces the mycotoxin tenuazonic acid. This fungus can have serious economic impact on sorghum production. Here, we report the draft genome sequence of *E. sorghinum* (USPMTOX48).

## GENOME ANNOUNCEMENT

*Epicoccum* *sorghinum* (Sacc.) (also known as *Phoma sorghina*) (1) is one of the most important fungi in the grain-mold complex in sorghum (2). The presence of this pathogen in sorghum results in significant economic losses due to reduced crop yields, seed viability, and kernel weight (3). This fungus produces tenuazonic acid (TA), a mycotoxin that produces acute toxicity to organisms and therefore prevents the consumption of sorghum grains as food and feed (4, 5). The draft genome of this fungus has genes involved in the TA pathway.

To begin to access the genetic mechanism of tenuazonic acid production in *E. sorghinum*, we report the draft genome sequence of *Epicoccum sorghinum* strain USPMTOX48, which was recovered from contaminated sorghum grains (*Sorghum* *bicolor* [L.] Moench cv. DKB 550) cultured in Votuporanga, Brazil, in 2013. A polyphasic approach consisting of molecular and morphological characterization was performed for species identification (1). For sequencing analysis, genomic DNA was extracted using the Easy-DNA kit (Invitrogen, USA) and used to generate a short-insert paired-end library on an Illumina HiSeq 2000 instrument.

The library generated 58,194,228 reads (read lengths, 101 bp) totaling 5,878 Mbp (176× genome coverage). Raw data underwent quality control using FaQCs, which trimmed and filtered the reads (6, 7). The resulting data were assembled with IDBA_UD (8) and Velvet (9). Consensus sequences of both assemblies were computationally shredded and merged with Phrap (10, 11). The genome assembly consisted of 391 contigs (>1 kb); the estimated genome size is 33.4 Mbp, and the G+C content is 52%.

Gene annotation was carried out using the MAKER2 training and annotation pipeline (12). Briefly, repeated genomic regions were masked using RepeatMasker (http://www.repeatmasker.org). Genes were then modeled by combining several gene annotations methods as inputs into MAKER2, namely: (i) BLASTx alignment of proteins of a related species, *Phoma tracheiphila*; (ii) Augustus (13) *ab initio* gene models trained on the gene structures of the fungal Benchmarking Universal Single-Copy Orthologs (BUSCO) (14); (iii) SNAP (15) *ab initio* models trained on Hidden Markov Models of the CEGMA core eukaryotic genes (16); and (iv) Genemark-ES *ab initio* gene models (17). BUSCO quality analysis of the output gene annotations resulted in a high-quality gene annotation. The output resulted in MAKER calling a total of 9,495 genes. The average gene length, the mean exon length, and the mean intron length were determined to be 1,658 bp, 574 bp, and 84 bp, respectively. Functional annotation of the 9,495 genes was performed by InterProScan 5 (18) and BLASTp searches against the UniProt (UniProt Consortium) protein blast database.

In agreement with the capability of *E. sorghinum* to produce TA, we found an identical domain of the TA biosynthetic gene described from *Magnaporthe oryzae* genome (19). TAS1 is a nonribosomal peptide synthetase (NRPS)-polyketide synthase (PKS) hybrid enzyme with a C-A-PCP-KS domain organization. The TAS1 identified in the *E. sorghinum* genome was highly conserved. This genomic information will contribute to a better understanding of the TA biosynthetic pathways and its regulatory mechanisms.

### Accession number(s).

The draft genome sequence of *Epicoccum sorghinum* (USPMTOX48) has been deposited at DDBJ/EMBL/GenBank under the accession no. MIEO00000000. The version described in this paper is version MIEO01000000.
